# Specific Mycoparasite-*Fusarium Graminearum* Molecular Signatures in Germinating Seeds Disabled Fusarium Head Blight Pathogen’s Infection

**DOI:** 10.3390/ijms22052461

**Published:** 2021-02-28

**Authors:** Seon Hwa Kim, Rachid Lahlali, Chithra Karunakaran, Vladimir Vujanovic

**Affiliations:** 1 Department of Food and Bioproduct Sciences, University of Saskatchewan, 51 Campus Drive, Saskatoon, SK S7N 5A8, Canada; sek049@mail.usask.ca; 2 Canadian Light Source, 44 Innovation Blvd, Saskatoon, SK S7N 2V3, Canada; rlahlali@enameknes.ac.ma (R.L.); chithra.karunakaran@lightsource.ca (C.K.); 3 Department of Plant Protection, Phytopathology Unit, Ecole Nationale d’Agriculture de Meknès, BP/S 40, Meknès 50001, Morocco

**Keywords:** seeds, fourier transform infrared (FTIR), interactome, mycoparasitism, *Fusarium graminearum*, fusarium head blight (FHB), biocontrol, *Sphaerodes mycoparasitica*

## Abstract

Advances in Infrared (IR) spectroscopies have entered a new era of research with applications in phytobiome, plant microbiome and health. *Fusarium graminearum* 3-ADON is the most aggressive mycotoxigenic chemotype causing Fusarium head blight (FHB) in cereals; while *Sphaerodes mycoparasitica* is the specific *Fusarium* mycoparasite with biotrophic lifestyle discovered in cereal seeds and roots. Fourier transform infrared (FTIR) spectroscopy analyses depicted shifts in the spectral peaks related to mycoparasitism mainly within the region of proteins, lipids, also indicating a link between carbohydrates and protein regions, involving potential phenolic compounds. Especially, *S. mycoparasitica* contributes to significant changes in lipid region 3050–2800 cm^−1^, while in the protein region, an increasing trend was observed for the peaks 1655–1638 cm^−1^ (amide I) and 1549–1548 cm^−1^ (amide II) with changes in indicative protein secondary structures. Besides, the peak extending on the region 1520–1500 cm^−1^ insinuates a presence of aromatic compounds in presence of mycoparasite on the *F. graminearum* root sample. Monitoring shift in improved seed germination, fungus-fungus interface through scanning electron microscopy (SEM) and confocal laser scanning microscopy (CLSM), and FTIR molecular signatures combined with principal component analysis (PCA) proved useful tools to detect an early mycoparasitism as a vital asset of the preventive biocontrol strategy against plant pathogens.

## 1. Introduction

Across plants, host-associated microbiomes play a vital role in shaping host defense via regulating expressions of resistance genes and plant health. Seed or grain, known as kernel in wheat, is considered as a plant reproductive unit that harbors a core microbiome essential for seed germination, plant growth and reproduction under stress [1]. Agrifood system is using elite seed lines for the staple crop establishment and production to maximize yield—the primary food source for the human population [2]. As such, seed is becoming a subject of increasing interest to an early control of fungal pathogens, molds and mycotoxins in crops leading to the creation of the “plant prenatal care” concept [3]. Seed harbors two distinct functional groups of fungal populations, named beneficial biocontrol and plant pathogenic agents. Healthy seeds are essential for the optimal crop growth and yield, but seed-borne pathogens such as *Fusarium* spp. may reduce seed vitality, germination, and cause damping-off disease of seedlings. In cereals, Fusarium head blight (FHB) caused by *Fusarium graminearum* (*F. graminearum*) can severely impact seed quality and limit the availability of seed [4]. In other hand, the fungal antagonists including mycoparasitic fungi [5] and mycoparasitism-related genes [6,7,8] have tremendous potential in controlling *Fusarium* and improving plant protection. Hence, the global food security research is seeking for mycoparasites microorganisms as ecofriendly biocontrol strategies to reduce *Fusarium* damages on seed and minimize mycotoxins in staple cereal foods [9]. The particular focus is on the suppression of *Fusarium* infectious process. The FHB disease curve starts during seed germination and progresses during anthesis, grain development and seed dispersion. The first is a critical developmental stage when an early biocontrol of seed-born *Fusarium* can be also protective to reduce mycotoxins in crops. The biocontrol approach-based on mycoparasitism is attracting more attention among agri-food, human and animal health scientists [10]. It is a promising tool to reduce the use of synthetic chemicals in staple crop by providing prenatal care to plant. 

Traditionally, phytoprotection is seeking for biocontrol solutions [11] deemed appropriate to manage seed-born *Fusarium* diseases and outbreaks while protecting the environment and food safety without genetically engineered plants. However, it is likely that the spectrum of studied mycoparasites against *Fusaria* and related FHB symptoms in cereals has been limited to *Trichoderma* and *Clonostachys* fungal generalists, although these non-specific mycoparasites have not yet achieved an economically and environmentally sustainable control perspective [12]. Recently, biocontrol science shifted from these non-specific to specific biocontrol fungal agents such as mycoparasitic *Ampelomyces* against powdery mildew (Erysiphales) and *Sphaerodes* against *Fusaria* pathogens and mycotoxins in cereal grain [13]. The term mycoparasitic lifestyle can be defined as a parasitic fungus-fungus relationship, in which the mycoparasite interacts with the fungus-host cell to perform its biological cycle in interaction with plant-host tissue [14]; the interaction involves several signaling pathways to ensure a mycoparasite’s biocontrol effectiveness.

*Sphaerodes mycoparasitica* is owning its biotrophic mycoparasitic lifestyle on parasitic *Fusarium* hosts, while its mechanism of mycoparasitism at molecular level has not yet been established. As a result, molecular studies of these biocontrol fungus as other obligate mycoparasites (e.g., *Ampelomyces*) are seriously lagging behind those of necrotrophic biocontrol generalists (e.g., *Clonostachys, Trichoderma*). In addition to biocontrol, *S. mycoparasitica* proved endosymbiotic plant growth promoting ability in enhancing wheat seed germination [14]. However, the endophytic mycoparasitism is often regulated by a combination of intrinsic and extrinsic factors. Intrinsic-genomic, transcriptomic and metabolomic factors may have an important role in the specific biocontrol of the plant pathogenic *Fusarium* in wheat seed [1,12]. Recent Fourier transform infrared (FTIR) work has drawn fascinating connections between fungal endophyte-wheat seed interaction changes under stress-drought conditions shedding more light on biological wheat seed stratification and mycovitality [15]. The mycovitality (vitalitas = *‘*life-force’) defines plant performance in a *continuum* with mycotrophy (trophe = ‘nutrition’). Through mycovitality (endophyte-seed association), endosymbiotic inoculants, via coleorhiza-driven seed stratification, were shown to regulate wheat’s (*Triticeae*) phytohormones, enhancing crop hardiness to environmental stressors. Previous results also demonstrated the ability of *S. mycoparasitica* to promote wheat seed germination under in vitro conditions. This prenatal care concept taking into consideration seed-related plant growth promotion (PGP) and biocontrol has piqued the interest of the global food security scientists, as the synergy of the two mechanisms in endophytic mycoparasites is remaining unexplored. It’s particularly true in terms of seed-health potential linked to the mycoparasite-*Fusarium* interacting molecules during early events of *Fusarium* suppression on seed germinants. To accurately measure the practical outcomes of the mycoparasitism, one should consider the effectiveness of mycoparasite relative to cell-to-cell interaction between the mycoparasite—the plant pathogenic host and—the plant host at the tissue and molecular level. Hence, we hypothesized that *S. mycoparasitica* positively shapes a profile of plant protective molecular signatures in germinating cereal seed-radicle cells, thus suppressing FHB pathogen in its early stage of host infection. 

The biocontrol agents that regulate wheat kernel metabolites against *Fusarium graminearum* are still poorly characterized. In the past decade, several metabolomics attempts have been made to decipher the chemical defense that cereals employ to counteract *F. graminearum* [16,17]. Although some major classes of metabolites have been highlighted (e.g., amino acids; carbohydrates; fatty acids; organic acids, phenylpropanoids and terpenoids) [17,18], the knowledge remains partial when comes to the integrated network of chemicals in cereal kernels stimulated by biological control agents [19]. Recently, FTIR revealed the beneficial fungus-dependent components changes in kernel through germinating kernel-fungus interaction under abiotic stress [15] opening new horizons for research on the kernel-beneficial fungus chemical profile or orchestrated metabolites to resist to *F. graminearum* as a biotic stress.

The FTIR spectroscopy has demonstrated its usefulness as a quick, reliable and valuable technique to depict the presence of functional groups of compounds identification [20,21] in heterogeneous biological samples including grain, pollen, coleorhiza, plants, soils and microorganisms [15,21,22,23]. Therefore, the scanning electron microscopy (SEM) and confocal laser scanning microscopy (CLSM) coupled with FTIR (mid-infrared) spectroscopy analytical methods were employed to elucidate seed structural tissue and metabolome responses to *F. graminearum* colonization under pathogen conductive (normal) versus suppressive (mycoparasite) interactive conditions. Quantifying metabolic shift in the seed tissues of *Triticum* host germinant is essential for understanding the mycoparasitic effect of *S. mycoparasitica* during seed germination together with shifts in interactome profile associated to reduce *Fusarium* infection/attack on seed germinants. The FTIR spectroscopy allows monitoring of compositional changes in cells/tissues (components) by detecting functional groups of different compounds. 

Investigation of FTIR biochemical composition changes in wheat seed radicles (primary roots) may predict plant response to the capacity of *S. mycoparasitica* against *F. graminearum* 3-ADON. Further, mapping the expression of key signatory molecules within tripartite mycoparasite-*Fusarium*-plant host interactions may further elucidate the mechanisms of mycoparasitism by which mycoparasite improves host traits and control *Fusarium* to secure a prenatal health in cereal host. The overall goal is to generate fundamental knowledge on tripartite seed endosymbiosis and mycoparasitism related interactome traits, thus creating a tool to depict early biochemical signatures for improved crop stress resilience to plant pathogens and mycotoxins.

## 2. Results and Discussion

### 2.1. In Vitro Wheat Seed Germination and Growth

**Figure 1:**
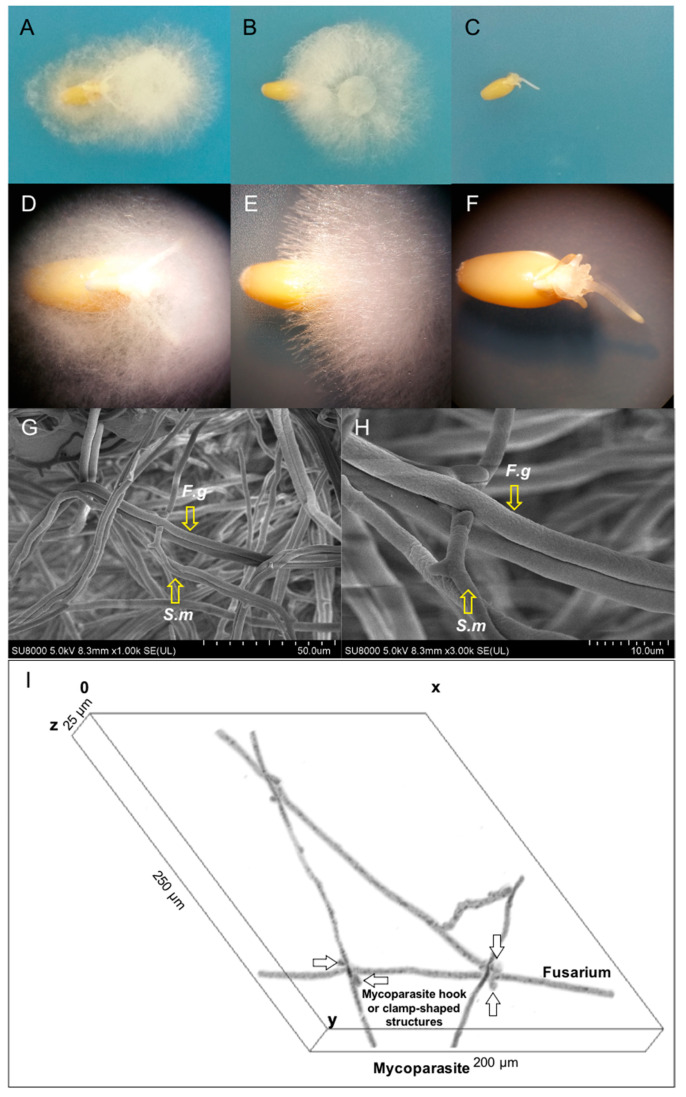
In vitro root and coleoptile growth of 2 days-old wheat kernel (seed) germinant (*Triticum turgidum* L. ssp. *durum (*Desf.)) under the different biotic conditions. (**A**,**D**), the seed surrounded by *S. mycoparasitica* (left side mycelium) against *F. graminearum* (right side mycelium); (**B**,**E**), the seed faced with *F. graminearum*; (**C**,**F**), the non-treated seed (control). The coleoptile and radicle of the mycoparasite treatment against *F. graminearum* were clearly visible compared with that of the *Fusarium* treatment. (**G**) (scale: 50 µm) and (**H**) (scale: 10 µm), SEM of the fungal interface between the mycoparasite (*S. mycoparasitica*) and *F. graminearum* interactive hyphae. (**I**). CLSM-tridimensional (X = 200 µm × Y = 250 µm × Z = 25 µm) view of the “triangular” *S. mycoparasitica-F. graminearum* hyphal network in which mycoparasitic hook- or clamp-shaped infective structure (arrows) attacks on *Fusarium* hyphae.

*S. mycoparasitica* showed the protective effect on the seed by maintaining seed vigor and suppressing the growth of *F. graminearum* compared to the seed of the *F. graminearum* treatment only (Figure 1A–F). There was no symptomatic, necrotic root or coleoptile rot, samples associated with *Fusarium* infection observed in the seed surrounded by *S. mycoparasitica* seed during 2-day incubation. The control seeds were also healthy. 

**Figure 2:**
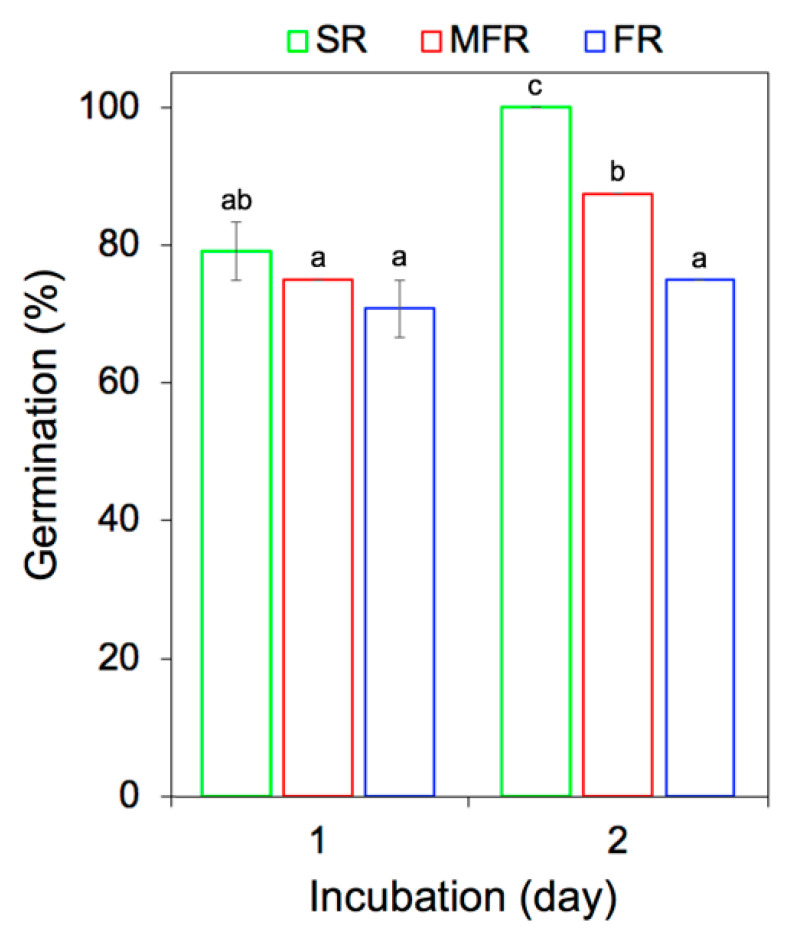
In vitro seed germination (%) under the different biotic conditions. The seed surrounded by the mycoparasite *S. mycoparasitica* against *F. graminearum* (MFR, red square line); the seed infected by *F. graminearum* (FR, blue square line); the non-treated or non-infected seed (SR, green square line). The germination (%) was calculated by the ratio of the number of germinated seeds for each treatment over the number of germinated seeds at day 2 control multiplied by 100. The presented bars are means of three sets of germination (%) with standard errors. The different letters (a–c) indicate statistically significant differences (*p* < 0.05) according to Tukey’s honestly significant difference test.

**Figure 3:**
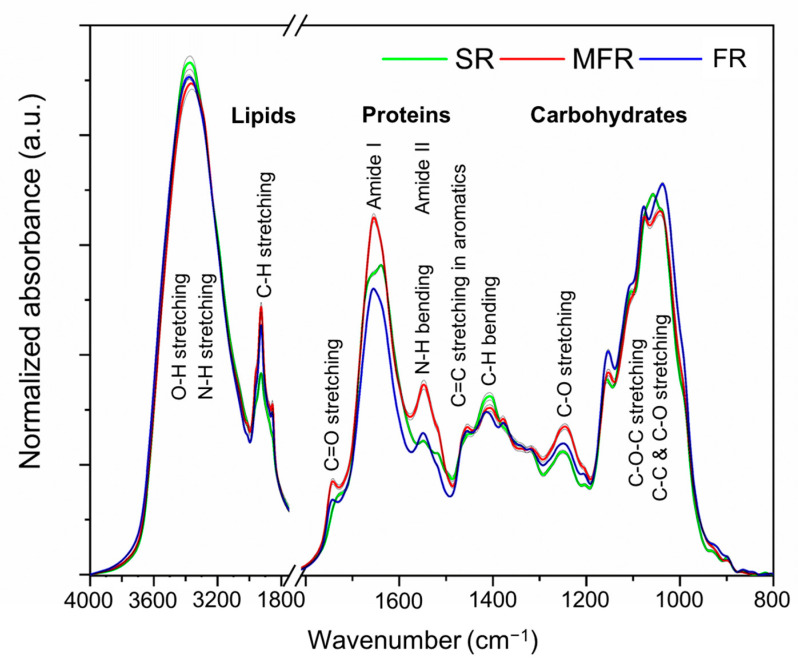
The vector normalized and average FTIR spectra of the root samples collected from germinating seeds (two-day-old) surrounded by *S. mycoparasitica* against *F. graminearum* (MFR, red line); the seed infected by *F. graminearum* (FR, blue line); the non-treated or non-infected seed (SR, green line). Data are means of vector normalized 6 spectra for each treatment and standard errors are shown as black lines.

The protective effect of the mycoparasite for the seed could be derived from the mycoparasitic interaction with the host for which evidently, *S. mycoparasitica* and *F. graminearum* interactive hyphae were observed on SEM (Figure 1G,H). In Figure 1I, the CLSM-tridimensional view depicted *S. mycoparasitica* hyphal network—a formation of the protective “shield” surrounding *F. graminearum* hyphae. The fungus-fungus interface is formed ~1 mm distance from the seed surface; it is characterized by “triangular” *S. mycoparasitica-F. graminearum* hyphal network in which mycoparasitic hook- or clamp-shaped infective structure attacks on *Fusarium* hyphae. This early step in mycoparasitism allowed *S. mycoparasitica* to suppress the progress of *F. graminearum* pathogenic hyphae towards seed-host tissue. Furthermore, the epiphytic *S. mycoparasitica* hyphae on seed coat followed by endophytic colonization of seed germinant [1–2day] allowed promotion of seed germination and growth, respectively, as previously described by Vujanovic and Goh [14]. As shown in Figure 2, the germination (%) of the seed surrounded by *S. mycoparasitica* against *F. graminearum* was significantly higher (87.5%) than that (75%) of the seed faced by *F. graminearum* only at day 2. The non-treated seed (control) showed 100% germination. This shift in phenotypic and biological/behavioral traits in germinating seed, induced by the mycoparasite on the *F. graminearum* root samples (Figure 1), was also analyzed by its contribution to the associated changes in FTIR molecular signatures (Figure 3). 

### 2.2. FTIR Spectra 

#### 2.2.1. Implication of Biochemical Changes in the Root Composition of Wheat Seed 

FTIR spectroscopy profile depicted considerable differences of the germinating seed-root samples when treated with fungi (FR—virulent *F. graminearum* 3-ADON alone); (MFR- mycoparasitic *S. mycoparasitica* + virulent *F. graminearum* 3-ADON) compared to the samples of the roots (SR) without any fungal inoculation (control). The baseline corrected normalized FTIR spectra of the root samples in the range 4000–800 wavenumber (cm^−1^) are shown in Figure 3. The biochemical FTIR profiles of the root samples are represented by characteristic peaks for lipids, proteins, and carbohydrates mainly in the spectral regions 3050–2800 cm^−1^, 1800–1200 cm^−1^, and 1200–800 cm^−1^, respectively. The tentative assignment of the characteristic peaks representing different functional groups of potentially possible components or compounds were summarized in Table 1. It is important note that the peak position (at wavenumber), intensity, and shape were not accurately matched with the data of the references mainly due to the different samples affecting the vibrational modes, but the major and typical characteristics for the peaks were applied to the tentative assignment for this study. For the sample comparisons, the term “intensity” was referred as a semiquantitative manner.

On the normalized spectra in Figure 3, the broad and strong peaks at about 3430–3300 cm^−1^ with a maximum range between 3379–3366 cm^−1^ were observed and assigned to NH stretching of amines from proteins and OH stretching of alcohols that might be derived from carbohydrates (including cellulose, hemicelluloses, starch, and fructans), glycoconjugates or glycomolecules (e.g., arabinogalactan proteins and extensins belonging to the superfamily of hydroxyproline-rich glycoproteins constituting approximately 10% of the dry weight of plant cell wall) [36], and metabolites related to root formation/development and plant defense responses. The peak at 3372 cm^−1^ in the control (SR) was more intense than that of fungal treatments (FR > MFR; the integrated absorption peak for MFR was significantly reduced shown in Table 2) and slightly shifted in both MFR (to 3366 cm^−1^) and FR (to 3379 cm^−1^), implying that there might be changes in the hydrogen bonds among the root components (including potentially proteins, carbohydrates, and glycoconjugates) or variations in combinations of root components during the interactions with the different fungi which might be linked to plant interactive or defense responses. 

The observed peaks in the region between 3050–2800 cm^−1^ were assigned to CH_2_/CH_3_ stretching of mainly lipids which broadly could include all components with aliphatic portions such as (membrane) lipids, lipid-derived molecules, fatty acids, and waxes (e.g., suberin and/or cuticular components) [37]: -CH stretching at 3014 cm^−1^ acyl lipids; asymmetric and symmetric CH_3_ stretching of mainly lipids at 2961 cm^−1^ and 2873 cm^−1^; asymmetric and symmetric CH_2_ stretching of mainly lipids at 2927–2924 cm^−1^ and 2856–2853 cm^−1^; CH_2_/CH_3_ bending (deformation) of mainly lipids (alkanes) and proteins at 1456–1453 cm^−1^; C = O stretching of carbonyl compounds at 1744–1700 cm^−1^ which may contain fatty acids, phospholipids, phytosterol esters, alkyl esters (related to pectins, hemicelluloses, and lignins), and any other aliphatic and aromatic carbonyl compounds. In particular, the ester linkage may be derived from the carboxylic groups of the *p*-coumaric- and ferulic acids for the hemicellulose and/or lignin [38]. The intensity of the peaks in this region (3050–2800 cm^−1^) was increased significantly in the fungal treatments (more prominent in MFR than FR, but the integrated peak areas of MFR and FR for this region were not significantly different) when compared with the control (Table 2), which may reflect increased production of lipids such as phospholipids, glycolipids (mainly galactolipids), and sphingolipids (mainly composed of glycosyl-inositolphosphoceramides) located in the plasma membrane and lipid-derived metabolites of the plant host during plant-microbe interactions related to plant immune signaling [39,40,41]. In addition, in the case of the peak at 3014 cm^−1^, the shape of the peak was relatively noticeable in MFR than FR compared to the control (SR), although the observed peak was weak which may indicate the presence of unsaturated fatty acid chains (derived from the fungi directly) [29]. 

The presence of carbonyl compounds (e.g., pectins or pectic polysaccharides which are ubiquitous polysaccharides in plants; usually more found in roots than leaves) was expected due to C = O stretching. However, the control showed no clear peak at this region. Importantly, there was the obvious peak at 1744 cm^−1^ for MFR with the outstanding appearance and at 1743 cm^−1^ for FR (the integrated absorption peak of MFR was significantly higher than that of FR and SR shown in Table 2), implying the heterogeneity of the chemical structures of the carbonyl compounds which could be affected by conjugation with the ester single-bonded oxygen and may extend the variety of possible root cellular components including fatty acids, aliphatic and aromatic carbonyl compounds, hemicelluloses, and lignins, not just subjective to the pectins. It was supportive that the esterification (%) used to evaluate the degree of esterification to which the ratio of the peak areas for C = O stretching over the sum of the peak areas for C=O stretching and amide I was supposed to be proportional [21] was significantly higher in MFR and FR than in SR (Table 2).

Apart from the peaks for OH and NH stretching (around 3430–3300cm^−1^), the relatively prominent peak was observed at 1655–1638 cm^−1^ assigned to protein amide I attributable to (C = O stretching from –CO-NH), along with the peak at 1549–1548 cm^−1^ assigned to amide II (attributable to N-H bending and C-N stretching) and the peak (at 1319–1318 cm^−1^) assigned to amide III (resulted from more complex vibrational modes than amide I and II) [32]. The intensity of the three peaks derived from proteins in MFR was higher than that of SR or FR (MFR > SR > FR for amide I; MFR > FR > SR for amide II; MFR > FR > SR for amide III). Particularly, the integrated absorption peaks for the amide I and II were significantly greater in MFR than in SR or FR (Table 2), implying the probable proteins were more affected by the mycoparasite. Noticeably, the control showed a different peak shape with the peak center shifted to 1638 cm^−1^, compared to fungal treatments. This difference in the peak shape may reveal the appearance of different protein secondary structures such as *α*-helices (at 1655), *β*-turns (at 1675), and *β*-sheets (at 1633) [42,43]. It seemed that the control tended to have higher *β*-sheets (at 1633) among the possible protein secondary structures, whereas the fungal treatments tended to have higher *α*-helices (at 1655) of them. The variation of protein secondary structures corresponded to the previous findings of Lahlali et al. [24] who reported that the susceptible cultivar showed an increase in *α*-helices and a decrease in *β*-sheets responding to the pathogen, which may imply plant resistance responses to stress through regulation of phenylpropanoid and lignin biosynthesis [24]. 

With relation to lignin, it seemed that the control sample had the relatively noticeable peak shape at 1518 cm^−1^ compared to fungal treatments. MFR showed a broad shoulder shape at this region (1520–1500 cm^−1^) which may be attributable to C = C stretching of aromatic rings in lignin [24], ferulic acid [28], and an aromatic ring containing amino acids (e.g., tyrosine) [29]. At this region, the integrated absorption peak was higher in MFR than in SR or FR (Table 2). Presumably, the region between 1520–1300^−1^ may contain any other aromatic compounds due to the complex absorption profiles. Especially, MFR tended to show the extended absorption peaks at 1516 and 1379 cm^−1^ among the treatments indicating those peaks are indicative only for the mycoparasite treatment as potential signatory molecules.

The peak at 1250–1246 cm^−1^ (C-O stretching) was mainly assigned as hemicellulose [24] and might be supported partly by asymmetric P = O stretching (of PO^2-^) for phospholipids and nucleic acids. Mycoparasite treatment showed an increase in the intensity of the peak compared with the control and *Fusarium* treatment, which implies a more presence of hemicellulose, phospholipids, and nucleic acids. The tendency for the increased hemicellulose during the interaction with plant pathogens was reported for the susceptible and resistant plants (canola roots) related to plant resistance responses [24]. Therefore, it was assumed that hemicelluloses with some potential compounds other than lignins are considerably involved in plant resistance derived by the mycoparasite in our study.

Besides those prominent peaks, the second prominent peak was observed at 1058–1036 cm^−1^ attributable to C-O-C symmetrical stretching, C-C, C-O stretching or C-OH bending of primary and secondary alcohols such as carbohydrates (mainly cellulose, hemicellulose). The extended region between 1200–900 cm^−1^ may indicate very complex contributions from different cell wall components (cellulose and hemicellulose) such as arabinoxylans (especially, main hemicellulose in wheat and barley) and xyloglucan (generally known as abundant hemicellulose in all plants) for which, the peak at 1157–1153 cm^−1^ may be responsible for mainly C-O-C asymmetric stretching and C-O stretching, as well as, cellular components such as phospholipids and nucleic acids which may be partly supported by the peak at 1077–1076 cm^−1^ along with 1250–1246 cm^−1^ attributable to symmetric and asymmetric stretching of P = O stretching (PO^2-^), respectively. In this region, MFR showed a relatively reduced intensity of the peak compared with FR and SR, implying that mycoparasite treatment possesses less cellulose. 

#### 2.2.2. Principal Component Analysis (PCA)

The principal component analysis (PCA) was performed to classify the data (FTIR spectra) among the treatments. 

**Figure 4:**
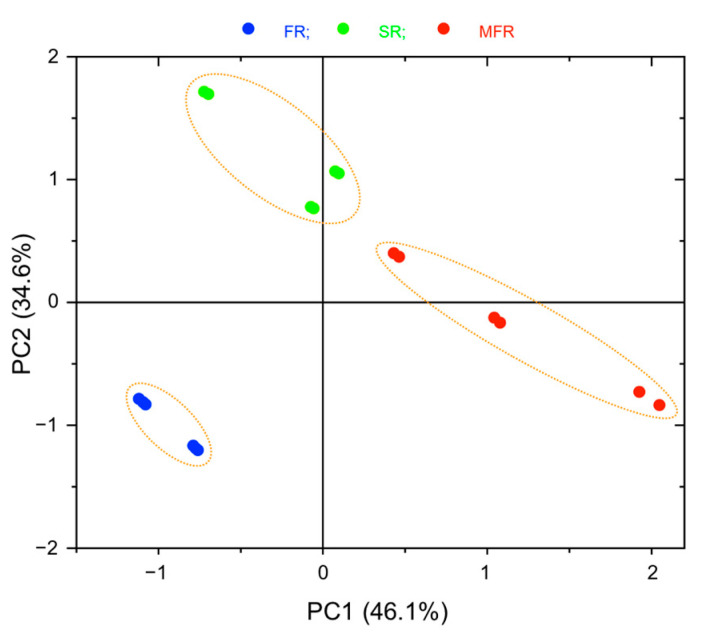
The scatter score plot based on principal component analysis of the FTIR spectra of the root samples. Each of the roots (*n* = 3; each pellet was measured two times) was collected from the seed surrounded by *S. mycoparasitica* against *F. graminearum* (MFR, red dot); the seed infected by *F. graminearum* (FR, blue dot); the non-treated or non-infected seed (SR, green dot).

The two-dimensional score plot of the PCA in Figure 4 indicated the clear separations among the root samples between the treatments with the fungi and the control. The PC1 and the PC2 explained 80.7% of the total spectral variance. The PC1 axis clearly distinguished MFR as a separate cluster on the positive side of PC1 from FR on the negative side of PC1, which indicates the significant difference of biochemical components depending on the presence of mycoparasite and the effect of mycoparasitism. The control (SR) showed the tendency to spread along the PC1 axis with a tilt to the positive side of PC2. The PC2 axis allowed the separation of SR clustered on the positive side of PC2, from FR clustered on the negative side of PC2. The MFR was scattered near the PC2 axis. 

**Figure 5:**
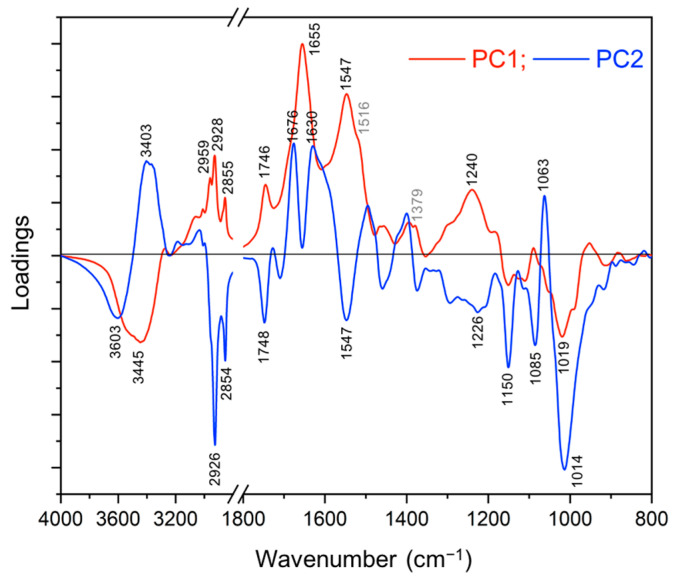
Loadings plot (PC1 & PC2) of the root samples based on the FTIR spectra. Each point in the plot represents the projection of a spectrum in the PC1–PC2 space.

The loadings plot of PC1 and PC2 are shown in Figure 5. In the region between 4000–3200 cm^−1^, the negative influence of PC1 loadings had one peak at 3445 cm^−1^, whereas the positive influence of PC1 had no peak. For PC2 loadings, the positive influence and negative influence were observed at 3403 and 3603 cm^−1^, respectively. These peaks may be partly supportive to differentiate roots (samples) due to the variations of possible molecules including carbohydrates, glycoconjugates, and proteins. 

For the lipid region between 3050–2800 cm^−1^, the three peaks at 2959 cm^−1^ (asymmetric CH_3_ stretching), 2928 cm^−1^ (asymmetric CH_2_ stretching), and 2855 cm^−1^ (symmetric CH_2_ stretching) on the positive influence of PC1 loadings are supportive to distinguish MFR from FR. Further, the negative influence of PC2 loadings shown at 2926 cm^−1^ (asymmetric CH_2_ stretching) and 2854 cm^−1^ (symmetric CH_2_ stretching) clearly differentiated FR from SR. Therefore, the increase in the lipid region of FR and MFR may imply that biotic factors such as *F. graminearum* and *S. mycoparasitica* (more prominent) contribute to the content of lipids on roots directly or indirectly. 

For the region between 1800–800 cm^−1^, the positive influence of PC1 loadings had peaks at 1746, 1655, 1547, and 1240 cm^−1^ which were assigned to carbonyl compounds (partly pectin), protein amide I (*α*-helices), amide II, and (mostly) hemicellulose, respectively, which were supportive to indicate these possible components were much more dominant in MFR (the mycoparasite treated *Fusarium* samples) than FR, of which, variation of protein composition was mainly responsible for the differentiation, significantly. The negative influence of PC1 loadings was shown at 1019 cm^−1^. 

The positive influence of PC2 loadings had three peaks at 1676, 1630 cm^−1^ assigned to amide I (*β*-turns and *β*-sheets), and 1063 cm^−1^ (mostly might be related to cellulose and hemicellulose) which differentiated the control SR (outstanding) from the FR. The negative influence of PC2 loadings showed the peaks at 1748, 1547, 1226, 1150, 1085, and 1014 cm^−1^ which also contributed to the differentiation of FR from SR by the tentative assignments of 1748 cm^−1^ to carbonyl compounds; 1547 cm^−1^ to amide II; 1226, 1014 cm^−1^ to carbohydrate; 1150, 1085 cm^−1^ to phospholipids and nucleic acids. 

In regard to the peak at 1516 cm^−1^, which has been previously assigned to lignin according to Lahlali et al. [24], this peak at the near region (1520–1500 cm^−1^) could be derived from any other aromatic or phenolic compounds due to C = C stretching of aromatic rings, with contributions at 1379 cm^−1^ which may be due to overlapping C-OH deformation of phenols and CH bending in aromatics [44,45,46]. 

Taken together, those noticeable peaks (at 1746, 1655, 1547, 1240 cm^−1^) along with other detectable peaks (1516, 1379 cm^−1^) imply existence of important signatory molecules defining mycoparasitism within this tripartite interactome profile, which could be a good example to investigate the mycoparasite effects on *Fusarium* in the presence of plant. 

#### 2.2.3. Estimation of Protein Secondary Structures in Tripartite System 

**Figure 6:**
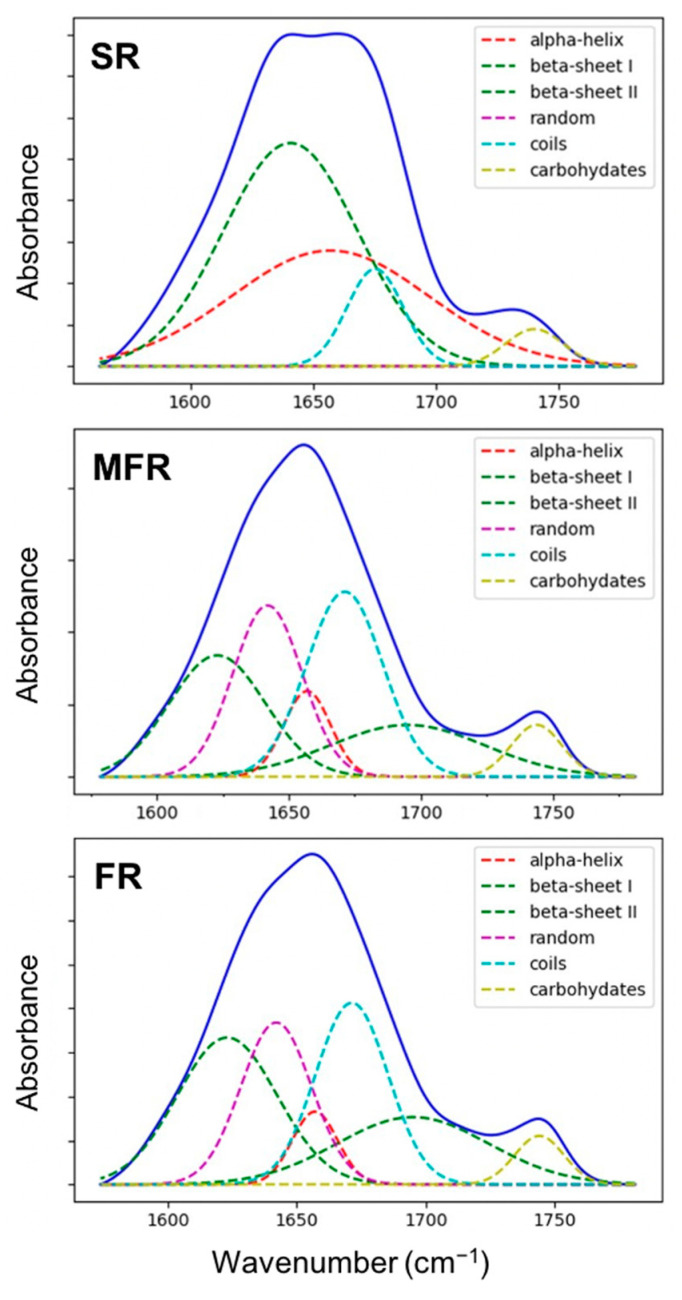
Curve fitting of the region (1580–1780 cm^−1^) for the root samples derived from each treatment (SR, the non-treated seed; MFR, the seeds surrounded by *S. mycoparasitica* against *F. graminearum*; FR, the seed infected by *F. graminearum*). Each spectrum is the average of 6 spectra.

The contributions of the protein secondary structures to the cell membrane are recognized as stress resilience factors in plants [21,47]. The protein secondary structural profiles of the germinating seed roots were estimated for each of the three biological (SR, FR, and MFR) samples. Figure 6 shows curve fitting results to α-helices, *β*-sheets, coils, and random. The fraction of each protein secondary structure associated with each of the biological treatments is listed in Table 3. Results indicate interesting differences in α-helical and β-sheets structures between biological treatments. The FTIR results depicted SR control with a relatively large amount (50.7%) of α-helices (1650–1658 cm^−1^). In germinating seed, primary root interaction with fungi decreased α-helices in host cells, which was less pronounced in MFR (4.2%) mycoparasite-*Fusarium* compared to FR pathogen (3.0%) samples. It is known that the α-helical structure proteins are particularly abundant in the cell membrane [47]. The *β*-sheets, consisted of β-sheet I (at 1630–1642 cm^−1^) and β-sheet II for turns and loops structures (at 1694–1682 cm^−1^), showed an inverse relationship with decreasing values from FR 61.2% to MRF 53.1% followed by SR 45.6%. The ratio between the α-helices: *β*-sheets parameter indicates the highest stress induced by FR (0.05) compared to MFR (0.08) which is a more adaptive endophytic interaction that protects germinating seed during mycoparasitism compared to aggressive-disease causing Fusarium plant pathogen [14]. 

Interestingly, the increased content of the α-helix band over *β*-sheet may suggest a higher sugar-protein presence in the matrix of MFR sample, such as trehalose (1657 cm^−1^), which may act as stabilizing stress compound. It could be produced by *Sphaerodes mycoparasitica* interacting with *Fusarium* pathogen in MFR, as the trehalose seemed to enhance the efficacy of the biocontrol agent against pathogenic molds [48]. According to FTIR study conducted by Imamura et al. [49], the trehalose embedded into protein matrix can also improve the cell membrane stability as measured by increased α-helix content (19%→45%), which was inversely correlated to *β*-sheet content (8%→4%), that aligned with results of this study. 

Lahlali et al. [21] have also found, in the pea pollen grain system, that the protein richness with α-helical structures is positively correlated with the protective effects on dehydration due to heat stress. Using FTIR spectroscopy, Belton et al. [50] study also proposed that the trehalose sugar molecules can markedly increase hydrogen-bonding contributions to the protein stability, which helps organisms exposed to stress in retaining cellular integrity. This effect is maximized when the water molecules are entrapped at the interface between the protein and the amorphous sugar matrix [50]. It seems that both water replacement and water entrapment are possible mechanisms mediated by hydrogen bonding, which occurs between the sugar and the protein [51]. 

Other secondary structural contributions such as coils and random protein structures were observed more in MFR and FR than in the control samples. It seems that under stressed conditions, there is a disordered protein structure with an increase in random and coils fraction, likely due to the more production of compatible solutes (sugars) in order to alleviate water stress [52]. This phenomenon was more pronounced in MFR treatment (42.7%) than in FR treatment (35.8%); suggesting that the endophyte fungus is contributing more to membrane integrity and stability under stress. However, the significance of those results is unclear, while no corresponding results were published for three-partite biological system interaction.

The carbonyl secondary structures (1760–1710 cm^−1^) usually provide information about the polar interfacial regions of membrane lipids or pectin [26,53] related to the biological membrane activity and structural flexibility. A shift in the absorption peaks in the carbonyl ester region was observed between MFR and FR indicating changes in the lipids, pectin compositions, or other carbonyl compounds between the two samples and relatively more stress induced by FR-*Fusarium* alone on germinating seed radicle compared to MFR (mycoparasite + *Fusarium*) and non-treated SR samples. The results in this study based on the biological stress response in wheat corroborate with abiotic stress response in other agricultural crops such as pea pollen [21], which merits further investigation.

Overall, it seems that modification of the protein secondary structure (PSS) based on α-helices: *β*-sheets fraction could be directly or indirectly related to the resistance of wheat tissue samples to combined biotic (plant pathogen) and abiotic (dehydration) stress. However, whether or not the PSS control dehydration during the host cell-fungus interaction or the sugar-trehalose embedded into protein matrix to improve cell membrane stability as a strategy to counter pathogen need to be further explored. 

Overall, the tripartite [mycoparasite-*Fusarium*-germinating seed] interaction results in the formation of a “triangular” *S. mycoparasitica-F. graminearum* hyphal network in which the infectious mycoparasitic hooked cells invade *Fusarium* hyphae for better control of *Fusarium* that coincides with a considerable shift in the protective host plant signatory molecules.

## 3. Materials and Methods

### 3.1. Plant and Fungal Materials

The present study was carried out using surface-sterilized seeds of AC Avonlea wheat (*Triticum turgidum* L. ssp. *durum* (Desf))—grown in a controlled CONVIRON^®^ PGR15 growth chamber (Controlled Environments Ltd., Winnipeg, MB, Canada) at the University of Saskatchewan and the surface-sterilized seeds were certified to be free of microbes [15]. The wheat seeds were surface-sterilized with the following steps as previously described [54,55]: immersing them in 95% ethanol for 10 s, rinsing in sterile distilled water for 10 s, submerging for 1 min in 5% sodium hypochlorite, and then rinsing three times in sterile distilled water. The Avonlea—durum variety was selected due to its low resistance to environmental stresses including FHB causing pathogens [56]. For this study, two fungal isolates from the Saskatchewan Microbial Collection and Database (SMCD), biocontrol SMCD 2220-01 strain of *S. mycoparasitica* Vujan. (2009) as a *Fusarium* specific mycoparasite [57,58], and SMCD 2243 *F. graminearum* Schwabe 3-ADON chemotype as a virulent plant pathogenic strain [13,59] were used. The fungi were grown on Potato Dextrose Agar (PDA) medium at 23 °C in darkness for 7 days and fresh cultures were used for in vitro assay. 

### 3.2. In Vitro Wheat Seed Germination Assay

*In vitro* wheat seed germination and early stage growth of germinating seeds were examined in the Biosafety-2 level lab at the University of Saskatchewan. The assessment was performed under the digital stereo microscope (DSM, Fisherbrand™ DC5420TH—Fisher Scientific, Ottawa, ON, Canada) (Figure 1A–F) microscope to investigate the effects of the mycoparasite *S. mycoparasitica* on the seed germination and changes in biochemical components of the wheat roots (radicles and seminar roots) against the plant pathogenic *F. graminearum* 3-ADON. The first treatment was composed of an agar plug (8 mm^2^) of *S. mycoparasitica* pre-inoculated on a PDA plate. After 2–day incubation of *S. mycoparasitica*, the pre-inoculated plug was gently removed from the plate to remain the mycelium of *S. mycoparasitica* on the plate. Then, a surface-sterilized seed of AC Avonlea was inoculated onto the mycelium of *S. mycoparasitica*. After 1–day incubation of the seed, an agar plug of *F. graminearum* was inoculated on the plate. The distance between *F. graminearum* and the seed surrounded by the mycelium of the mycoparasite (*S. mycoparasite*) was 0.5 cm (seed with *S. mycoparasite* against *F. graminearum*). The second treatment consisted only of the applied *F. graminearum*. A surface-sterilized seed was inoculated on a PDA plate. After 1–day incubation of the seed, an agar plug of *F. graminearum* was placed on the plate keeping the distance 0.5 cm away from the seed (seed against *F. graminearum*). As a non-treatment, a surface-sterilized seed was inoculated on a PDA plate (seed only). All the plates (9 cm diameter Petri dishes) were sealed with two layers of parafilm to maintain water activity (*a_w_* = 0.97) and kept at 23 °C in darkness during the incubation period. Each treatment includes 10 replicates. The 1 and 2-day-old seeds were counted for germination through direct or macroscopic observation. To obtain better quality of images, samples were transferred onto new Petri dishes and images were taken under the DSM. Percentage of seed germination (%) was calculated (Figure 2) with the following formula: (number of germinated seeds in particular treatment/number of germinated seeds in control) × 100; here, the control refers to the non-treated seed at day 2 [14]. For statistical analysis, three sets of germination (%) data were analyzed by one-way analysis of variance (ANOVA) with Tukey’s honestly significant difference (HSD) test (*p* < 0.05). The seed, radicle, and seminal root were then subjected to the sampling, microscopy, and FTIR spectroscopy analyses. 

### 3.3. Scanning Electron and Confocal Laser Scanning Microscopy

In order to observe the mycoparasitism on *Fusarium* host (Figure 1G–I), Scanning Electron Microscopy (SEM) and Confocal Laser Scanning Microscopy (CLSM) were applied by using an Ultra-High Resolution (1.0 nm) Scanning Electron Microscope (Hitachi SU-8010 FE-SEM) operating at 3kV and Confocal Laser Scanning Microscope (Leica TCS-SP5) equipped with Leica LAS AF imaging software at the University of Saskatchewan-WCVM Imaging Centre. For the SEM sample preparation, the interaction zone of the mycoparasite and Fusarium host on the agar (approximately 0.5 cm^2^) was dissected and subjected to protocols [60] with slight modifications. The excised blocks of agar with the mycelia or interacting hyphae were pre-fixed in 2% glutaraldehyde (GA) in 0.1M sodium cacodylate (NaCAC) at pH 7.2 for 1 h then rinsed with 0.1M NaCAC and stored at 4 °C prior to the osmium fixation. The pre-fixed samples were fixed with 1% osmium tetraoxide (OsO_4_) in 0.1M NaCAC for 1hr then, rinsed with sterile distilled water three times. Subsequently, the fixed samples were proceeded to following procedures; gradual dehydration with ethanol, substitution with amyl acetate, critical point drying on Polaron E3000, and coating with gold (10 nm thickness) using Q150T ES Quorum, turbo-pumped sputter coater [15]. For the CLSM, the mycoparasite and *Fusarium* host were co-cultured directly on a sterilized slide glass by providing 100 µL of potato dextrose broth for 2.5 days and then stained with 50 µL of 0.01% Lacto-fuchsin prior to the observation. The CLSM image was generated by using the open-source platform Fiji for biological-image analysis [61]. 

### 3.4. Preparation of the Root Samples for FTIR Spectroscopy

Germinating seed samples consisted of the radicle (primary root) and seminar roots collected by sterile blades under the DMS, gently washed with sterile distilled water, and lyophilized for 2 days to remove any residual moisture prior to sample preparation for FTIR spectroscopy (Canadian Light Source) using Bruker IFS 66V/S spectrometer) (Bruker Optics, Ettlingen, Germany) equipped with a KBr beam splitter at the mid-infrared beam. The freeze-dried pooled samples for each sample type were ground up to fine powders by using a mortar and a pestle to make the samples homogenous. The fine powder was ground (3 replicates for each sample type) with potassium bromide (KBr) to prepare pellets at the concentration of the sample in KBr of 1.3%. The prepared powders were pressurized under 55.16 Kilopascals (kPa) for 2 min to make transparent pellets (12 mm diameter) and kept in a desiccator until FTIR measurement [18]. The FTIR measurement was performed twice for each sample type. The Bruker IFS 66V/S spectrometer (Bruker Optics, Ettlingen, Germany) equipped with a KBr beam splitter at the mid-infrared beamline using the globar (silicon carbide) as the infrared source was used to collect FTIR spectra. Each IR spectrum was measured and recorded in the range of 4000–600 cm^−1^ at the resolution of 2 cm^−1^ and 64 scans per sample [24]. The number of replicates was 3 for each sample type. A KBr pellet without a sample was used as a blank for the background measurement.

### 3.5. FTIR Data Analysis 

FTIR data analysis and plotting were accomplished using OPUS (version 7.2, Bruker Optik GmbH, Bruker Optics Inc., Billerica, MA, USA) and Origin Pro (version 9.1, OriginLab Corporation, Northampton, MA, USA) programs. First, the pure KBr spectra were used to normalize FTIR spectra of the samples. The normalized FTIR spectra were baseline corrected using the rubber band correction (64 points), vector normalized, and averaged using the OPUS software (Figure 3). The FTIR peaks (Table 1) were determined using the Quick Peaks routine in Origin Pro with the settings of local maximum at 0% threshold height, no baseline, and area at Y = 0 [18]. The individual FTIR spectrum was integrated by the Peak Analyzer in Origin Pro with the settings of local maximum at 20% threshold height and Y = 0. The obtained (absorption) peak areas were analyzed by one-way ANOVA (*p* < 0.05) with Tukey’s HSD test (Table 2). The tentative assignment of the peaks representing different functional groups of compounds was performed in comparison with Web of Science published data and a largely accepted list of references [24,25,26,27,28,29,30,31,32,33,34,35], dataset, and information. The baseline corrected and vector normalized spectra were used for principal component analysis (PCA) to differentiate treatments and the scatter score and loading plots were shown in Figure 4 and Figure 5, respectively.

### 3.6. Protein Secondary Structure Analysis 

Once the spectral averages were calculated for each of the samples, the data was cropped between 1520 and 1780 cm^−1^ for further processing. A linear baseline was subtracted from the spectra before being fit to a 6 component Gaussian non-linear curve fitting model utilizing the Python package-Non-Linear Least-Squares Minimization and Curve-Fitting for Python, available online: https://lmfit.github.io/lmfit-py/ (Available online 5 December 2020). The peak areas of each component were calculated from the resulting parameter fits to yield percentage estimates of the various secondary structure motifs. This procedure was implemented using Quasar (Orange Spectroscopy [62]). The area under the entire band was considered 100% and each component after fitting was expressed as a percent fraction [21].

## 4. Conclusions

The wheat seed germination rate (%) was improved when the seed was protected by *S. mycoparasitica* against virulent *F. graminearum* 3-ADON chemotype. This implies that an early preventive biocontrol measure is possible to suppress the establishment of FHB pathogens in cereal crops. Further, the FTIR spectra showed biochemical changes in the root cells of the seed germinants under the tripartite *S. mycoparasitica*-*F. graminearum*-plant host interaction. Although the fungal treatment was associated with considerable shifts in the FTIR spectra, it is probable that the protective *S. mycoparasitica* induced multiple signatory molecules defining mycoparasitism. This finding indicates that *S. mycoparasitica* plays a crucial role in promoting wheat fitness by increasing defense responses of the germinating seed (root) against early *F. graminearum* colonization of the plant host. The spectral peaks related to mycoparasitism were noticeable mainly within the region of proteins and lipids, also indicating a link between carbohydrates and protein regions, involving potential phenolic compounds. A modification of the protein secondary structure, based on α-helices:*β*-sheets fraction, may further contribute to wheat cell membrane stability. Therefore, the effect of *S. mycoparasitica* on the wheat seed at a molecular level merits further examination to investigate bioactive compounds in providing prenatal care to plants against *F. graminearum* via endophytic mycoparasitism.

## Figures and Tables

**Table 1:** The tentative assignment of FTIR absorption peaks of wheat root samples-based on literature (Web of Science) data (see references). The wavenumbers presented in the table are the ranges for the mean values of the control and fungal treatments.

Wavenumber (cm^−1^)	Vibration Modes of Functional Groups	Probable Components	References
3430–3300	O-H stretching of Alcohols and carboxylic acids	Carbohydrates (cellulose, hemicelluloses) and Glycoconjugates	[24,25]
	N-H stretching of amide A	Proteins (amide A)	
2927–2924 2856–2853	C-H stretching of asymmetric and symmetric CH_2_	Lipids	[24,25]
1744–1700	C = O stretching of aldehyde, ketone, ester compounds, and carboxylic acid	Fatty acids, aliphatic and aromatic carbonyl compounds hemicelluloses, lignins, pectins	[24,25,26]
1655–1638	C = O stretching of -CO-NH	Proteins (amide Ⅰ)	[27]
1549–1548	N-H bending and C-N stretching	Proteins (amide Ⅱ)	[27]
1520–1500	C = C stretching of aromatic rings	Lignin related molecules; ferulic acid and aromatic ring containing compounds	[24,28,29]
1456–1453	C-H bending of CH_2_ and CH_3_	Proteins and lipids	[30,31]
1319–1318	C-N stretching and N-H deformation or more complex vibrations	Proteins (amide Ⅲ)	[32]
1250–1246	C-O stretching Asymmetric P = O stretching (of PO^2-^)	Hemicellulose, Phospholipids and nucleic acids	[24,27,30,33]
1157–1153 1077–1076	Mainly C-O-C asymmetric stretching and C-O stretching Symmetric stretching of PO^2-^	Mainly Cellulose, Phospholipids and nucleic acids	[24,27,30,33]
1058–1036	C-O-C symmetric stretching and C-C, C-O stretching or C-OH bending	Mainly Cellulose, hemicellulose	[24,27,34,35]

**Table 2:** Integrated absorption peaks for the FTIR spectra of the root samples collected from 2-day-old germinating seeds and esterification (%).

Peak Position (cm^−1^) with Relative Identity	Integrated Absorption Peak			
	Wavenumber Range (cm^−1^)	SR	MFR	FR
3430–3300 OH stretching and Amide A	3680–3000	19.58 ± 0.02 ^b^	19.18 ± 0.12 ^a^	19.54 ± 0.03 ^b^
2927–2924 2856–2853 Lipids	3000–2800	2.73 ± 0.06 ^a^	3.11 ± 0.06 ^b^	2.96 ± 0.02 ^b^
1744–1743 Carbonyl	1760–1720	0.23 ± 0.00 ^a^	0.30 ± 0.01 ^b^	0.24 ± 0.00 ^a^
1655–1638 Amide I	1700–1620	1.92 ± 0.01 ^b^	2.06 ± 0.03 ^c^	1.69 ± 0.00 ^a^
1549–1548 Amide II	1560–1530	0.35 ± 0.00 ^a^	0.49 ± 0.01 ^b^	0.37 ± 0.00 ^a^
1518–1516 C = C stretching of aromatic rings	1520–1500	0.21 ± 0.00 ^b^	0.23± 0.01 ^c^	0.18 ± 0.00 ^a^
Esterification (%) ^1^		10.70 ± 0.05 ^a^	12.56 ± 0.08 ^b^	12.47 ± 0.05 ^b^

SR, control seed not affected by fungi; MFR, seed surrounded by the mycoparasite against *Fusarium graminearum*; FR, seed infected by *Fusarium graminearum*. Each value is expressed as mean ± standard error (*n* = 6). Values with different letters (^a–c^) along the rows are significantly different (*p* < 0.05) by Tukey’s honestly significant difference test. ^1^ Esterification (%) was calculated by the peak (1744–1743) divided by the sum of peak areas for 1744–1743 and 1655–1638 and then expressed as a percentage.

**Table 3:** Secondary protein structures (expressed as %) of the root samples derived from each treatment (SR, the non-treated seed; MFR, the seeds surrounded by *S. mycoparasitica* against *F. graminearum*; FR, the seed infected by *F. graminearum*).

Protein Structure	Treatment			Ratio	
	SR	MFR	FR	SR:MFR	SR:FR
α-helices	50.7	4.2	3.0	12.07	16.9
*β*-sheets (I + II)	45.6	53.1	61.2	0.86	0.75
Coils	3.5	24.3	19.4	0.14	0.18
Random	0.2	18.4	16.4	0.01	0.02

Data were estimated from the average of 6 spectra per treatment. The percentage indicates a certain proportion of each protein structure to the total protein structures (100%).

## Data Availability

Not applicable.
